# Malaria illness mediated by anaemia lessens cognitive development in younger Ugandan children

**DOI:** 10.1186/s12936-016-1266-x

**Published:** 2016-04-14

**Authors:** Michael J. Boivin, Alla Sikorskii, Itziar Familiar-Lopez, Horacio Ruiseñor-Escudero, Mary Muhindo, James Kapisi, Victor Bigira, Judy K. Bass, Robert O. Opoka, Noeline Nakasujja, Moses Kamya, Grant Dorsey

**Affiliations:** Department of Psychiatry, Michigan State University, 965 Fee Road, Room A227, East Fee Hall, East Lansing, MI 48824 USA; Department of Statistics and Probability, Michigan State University, East Lansing, MI USA; Infectious Diseases Research Collaboration, Kampala, Uganda; Department of Mental Health, Johns Hopkins University Bloomberg School of Public Health, Baltimore, MD USA; Department of Paediatrics and Child Health, School of Medicine, Makerere University College of Health Sciences, Kampala, Uganda; Department of Psychiatry, School of Medicine, Makerere University College of Health Sciences, Kampala, Uganda; School of Medicine, Makerere University College of Health Sciences, Kampala, Uganda; Department of Medicine, San Francisco General Hospital, University of California, San Francisco, CA USA

## Abstract

**Background:**

Asymptomatic falciparum malaria is associated with poorer cognitive performance in African schoolchildren and intermittent preventive treatment of malaria improves cognitive outcomes. However, the developmental benefits of chemoprevention in early childhood are unknown. Early child development was evaluated as a major outcome in an open-label, randomized, clinical trial of anti-malarial chemoprevention in an area of intense, year-round transmission in Uganda.

**Methods:**

Infants were randomized to one of four treatment arms: no chemoprevention, daily trimethoprim–sulfamethoxazole, monthly sulfadoxine–pyrimethamine, or monthly dihydroartemisinin–piperaquine (DP), to be given between enrollment (4–6 mos) and 24 months of age. Number of malaria episodes, anaemia (Hb < 10) and neurodevelopment [Mullen Scales of Early Learning (MSEL)] were assessed at 2 years (N = 469) and at 3 years of age (N = 453); at enrollment 70 % were HIV-unexposed uninfected (HUU) and 30 % were HIV-exposed uninfected (HEU).

**Results:**

DP was highly protective against malaria and anaemia, although trial arm was not associated with MSEL outcomes. Across all treatment arms, episodes of malarial illness were negatively predictive of MSEL cognitive performance both at 2 and 3 years of age (*P* = 0.02). This relationship was mediated by episodes of anaemia. This regression model was stronger for the HEU than for the HUU cohort. Compared to HUU, HEU was significantly poorer on MSEL receptive language development irrespective of malaria and anaemia (*P* = 0.01).

**Conclusions:**

Malaria with anaemia and HIV exposure are significant risk factors for poor early childhood neurodevelopment in malaria-endemic areas in rural Africa. Because of this, comprehensive and cost/effective intervention is needed for malaria prevention in very young children in these settings.

## Background

The adverse effects of both cerebral malaria and severe malaria anaemia in preschool-age children has also been documented [1, 2]. However, uncomplicated malaria has also been associated with cognitive deficits in children. Vitor-Silva et al. concluded that uncomplicated malaria was associated with poorer school performance in the Brazilian Amazon [3]. In Sri Lanka in a series of studies by Fernando and colleagues, poorer academic performance and lower cognitive ability were associated with a higher number of malaria attacks in school children [4–7].

Intermittent prevention treatment (IPT) for malaria in schools and in communities has had cognitive and academic benefits in rural Gambia [8], western Kenya [9], Mali [10], and Zambia [11, 12], although one negative outcome was reported in a Kenyan study [13]. All these IPT clinical trials involved school-age children randomized by classrooms, schools or villages. The developmental benefits of IPT, randomized to individual children in early childhood, are unknown.

**Fig. 1:**
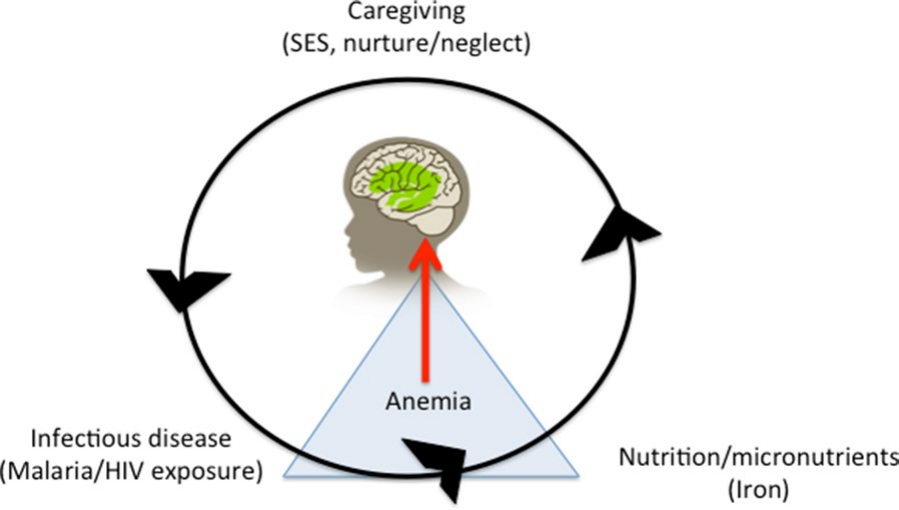
Brain development model as evaluated in the present study. The developing brain is measured by the Mullen Scales of Early Learning (MSEL) composite of the cognitive scales (visual reception, fine motor, receptive language, and expressive language). The measure of socio-economic status (SES) includes quality of caregiving (nurture/neglect) and physical growth (weight-for-age and height-for-age z scores) along with anaemia are partly the result of nutrition and micronutrients. Anaemia is also partly caused by the effects of infectious disease in early development, such as malaria and exposure to maternal HIV

The principal study questions are whether: (1) malaria illness and anaemia are related to poorer cognitive development in preschool-age children in an area of high malaria transmission; and, (2) whether perinatally HIV-exposed uninfected (HEU) children more vulnerable to the adverse effects of malarial illness on cognitive development, compared to HIV-unexposed uninfected (HUU) children. Figure 1 depicts the principal factors in the present study analyses (SES/Caregiving, Nutrition/Micronutrient Deficiencies and Anemia, Infectious Disease/Malaria/HIV) influencing brain development, which is measured by the MSEL composite score for the cognitive scales (visual reception, fine motor, receptive language, and expressive language).

## Methods

### Study site

This observational study of neurodevelopmental outcomes was embedded within a malaria chemoprevention randomized controlled trial (RCT) study conducted at the Tororo District Hospital near the Kenyan border in eastern Uganda [14, 15] (ClinicalTrials.gov number NCT00948896). Ethical approval was obtained from the Uganda National Council for Science and Technology, the Makerere University School of Medicine Research and Ethics Committee, and the Biomedical Institutional Review Board (IRB) of Michigan State University. Written informed consent was obtained from the principal caregiver (usually the biological mother) for each study child.

### Clinical trial of chemoprevention

All children still enrolled at 2 years of age in the RCT trials were eligible to be co-enrolled in the present study for MSEL assessment to take place at 2 and 3 years of age, except for those who had previously been hospitalized for severe malaria during the RCT study. For the RCT study, eligibility criteria for the HEU children included confirmed HIV-positive status of biological mother; confirmed negative HIV DNA PCR test of infant at time of enrolment; infant actively breastfeeding at the time of enrolment; residency within 30 km of the study clinic with no intention of moving outside the study area; agreement to come to the study clinic for any illness and to avoid medications outside the study protocol; provision of informed consent by parent/guardian; no history of allergy or sensitivity to any study drugs; absence of active medical problem requiring in-patient evaluation or chronic medical conditions requiring frequent attention; and absence of clinically significant electrocardiogram abnormalities, family history of long QT syndrome or current use of drugs that prolong the QT interval [15]. Only one eligible child was enrolled per household.

The HEU cohort mothers were on highly active antiretroviral therapy (HAART) during pregnancy and their study children were on nevirapine for 6 weeks and trimethoprim–sulfamethoxazole (TS) prophylaxis for the duration of breastfeeding, which is the standard of care in many African countries, including Uganda [16]. These HEU children were enrolled at about four to 5 months of age (median = 4.7 months) and monitored on a monthly basis. Six weeks following cessation of breastfeeding, a DNA PCR test was done for each participant and those who remained HIV uninfected were randomized to one of four regimens: no chemoprevention, daily TS, monthly sulfadoxine–pyrimethamine (SP), or monthly dihydroartemisinin–piperaquine (DP). For the HEU children cessation of breast feeding and subsequent randomization to RCT treatment arm ranged from six to 19 months of age (median = 9.7 months).

Eligibility criteria for the HUU children in the original RCT trial included the following [14]: (1) born to HIV-uninfected mothers, (2) residency within 30 km of the study clinic with no intention of moving outside the study area, (3) agreement to come to the study clinic for any illness and to avoid medications outside the study protocol, (4) provision of informed consent by parent/guardian, (5) no history of allergy or sensitivity to any study drugs, (6) absence of active medical problem requiring in-patient evaluation or chronic medical conditions requiring frequent attention, and (7) absence of clinically significant electrocardiogram (ECG) abnormalities, family history of long QT syndrome, and current use of drugs that prolong the QTc interval. Only one eligible child was enrolled per house.

The HUU study children in the original RCT trial were enrolled at 4–5 months of age (median = 5.6 months) and randomized to treatment arms at 6 months of age. Both the HEU and HUU children were followed for all their health care needs including monthly routine visits. Chemoprevention was discontinued at 2 years of age and children were followed for one additional year until they reached 3 years of age. The primary outcomes were the incidence of malaria and incidence of moderate-severe anaemia (haemoglobin <80 gm/dL).

At enrolment in the original RCT trial, caregivers were given two long-lasting, insecticide-treated bed nets (LLINs). Anthelminthic, iron sulfate and vitamin A were prescribed following Integrated Management of Childhood Illnesses guidelines. During follow-up, children who presented with a documented fever (tympanic temperature >38 °C) or a history of fever in the previous 24 h, had blood obtained by finger prick for a thick blood smear. Episodes of uncomplicated malaria were treated with standard doses of artemether–lumefantrine (AL). Episodes of complicated malaria or treatment failures occurring within 14 days of therapy were treated with quinine. Routine evaluations, including thick blood smears and assessment of adherence to LLINs and study drugs, were done monthly [15].

### Study procedure

**Fig. 2:**
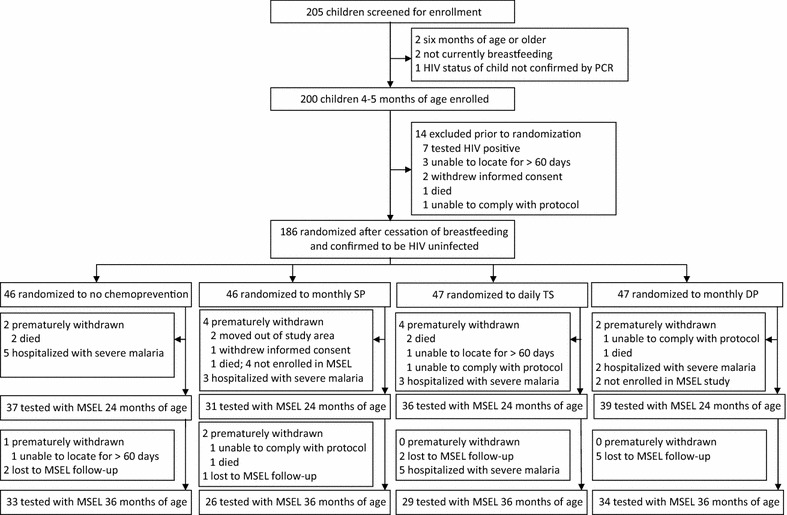
This is the CONSORT diagram showing the process whereby 186 perinatally HIV-exposed and uninfected children (HEU) at 4–5 months of age were enrolled in a randomized controlled trial (RCT) to evaluate the effects of four different malaria chemoprevention treatment arms on incidence of malaria illness and levels of anaemia. At 2 years of age, these children malaria chemoprevention was discontinued and children co-enrolled in the present study (N = 143) were evaluated with the Mullen Scales of Early Learning (MSEL). All RCT children continued to be monitored for malaria illness and anaemia until 3 years of age, at which time remaining eligible children were again evaluated with the MSEL (N = 122)

**Fig. 3:**
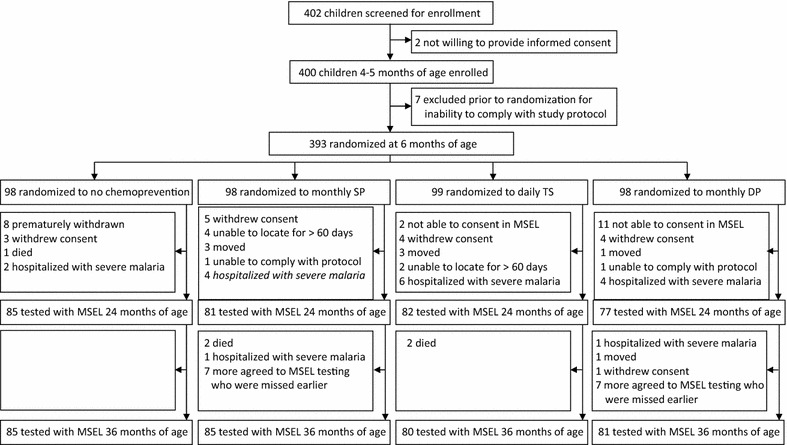
This is the CONSORT diagram showing the process whereby 393 perinatally HIV-unexposed and uninfected children (HUU) at 5–6 months of age were enrolled in a randomized controlled trial (RCT) to evaluate the effects of four different malaria chemoprevention treatment arms on incidence of malaria illness and levels of anaemia. At 2 years of age, these children malaria chemoprevention was discontinued and children co-enrolled in the present study (N = 325) were evaluated with the Mullen Scales of Early Learning (MSEL). All RCT children continued to be monitored for malaria illness and anaemia until 3 years of age, at which time remaining eligible children were again evaluated with the MSEL along with those co-enrolled between 2 and 3 years who were missed at the earlier 2-year of age assessment (N = 331)

Figures 2 and 3 are CONSORT diagrams depicting the manner in which 186 HEU and 393 HUU children were randomized into 4 malaria chemoprevention treatment arms. The published study findings for the HUU [14] and HEU [15] cohorts contain the detailed methodology for the malaria chemoprevention RCT within which the present neurodevelopment observational study was embedded. ITNs were distributed to all study participants at the time of enrollment. At the time of each monthly routine visit, parents/guardians were asked whether their child slept under an ITN the prior evening. At the time of treatment allocation, parents/guardians were given a 2 month supply of study drugs and a diary with dates for dosing and check-offs to indicate administration. Study drugs were resupplied to maintain a 2 month supply during clinic visits. At the time of each monthly routine visit, parents/guardians were asked how many doses of study drugs were given since the last routine visit and dates of administration were recorded.

At 2 years of age, all HEU and HUU children not lost to follow-up in the RCT trials, which had not been hospitalized with a history of severe malaria and with caregivers willing to participate, were co-enrolled into our observational study for neurodevelopmental assessment with the MSEL. Children who had been hospitalized with severe malaria were excluded from the present study because of previous findings documenting the relationship between severe malaria and MSEL performance [1, 17]. As can be seen in Fig. 2, 143 out of 186 HEU children were co-enrolled from the malaria chemoprevention RCT for MSEL testing at 2 years of age. In Fig. 3, 325 out of 393 HUU RCT children were co-enrolled for MSEL testing at 2 years of age. These combined totals for the RCT cohort samples ensured 80 % power for our MSEL neurodevelopmental outcomes based on prior work in Tororo [18].

Children’s neurodevelopment was assessed with the Mullen Scales of Early Learning (MSEL) twice; at 2 and again at 3 years of age. This assessment is intended for use in assessing children from birth to 68 months and provides assessment scores for the neurodevelopmental domains of gross motor (up to 33 months), and a composite score for the cognitive scales of visual reception, fine motor, receptive language, and expressive language. This test was designed to be more easily adaptable to resource-limited and cross-cultural settings than the Bayley Scales of Infant Development, considered to be the gold standard of such assessments [19]. At the same time, the MSEL has excellent correspondence validity with the Bayley scales [20]. The MSEL has been previously administered in local languages to evaluate the neurodevelopmental benefits of a caregiver training intervention with mothers of HEU children in Tororo [18]. The MSEL has also proven sensitive to the neurodevelopmental effects of severe malaria anaemia and cerebral malaria in Ugandan children [1, 17].

Research assistants administering the MSEL in the present study were all psychology Bachelor’s degree graduates of Makerere University fluent in the local languages who underwent 1 week of training on the test followed by a month of daily practice testing observation and participation. Following this training and practice period, the head of the assessment team at the study site observed the MSEL administration by the apprentice research assistant and approved the assistant for the assessment of study children contingent on good testing skills and satisfactory completion of an evaluation of MSEL testing skills as observed thereafter on a monthly basis (quality assurance program).

Socio-economic status (SES) was evaluated in the present study using a questionnaire read to the mother in the local language which asked a series of questions about the physical quality of the dwelling (materials and construction of floor, roof, walls, doors, windows, cooking facilities, toilet facilities), availability of water and plumbing, availability of electricity and sources of fuel for cooking/heating, material possessions such as common appliances, radio/TV, bicycle, dishes, furniture, and books. The mother was also asked a series of questions about the types of employment for pay for her and the father/husband or other adult household members, as well as domestic animals available to the household. The SES questionnaire asked about food security in the way of sources of meat, fish, poultry, agriculture and garden produce. Finally, the SES questionnaire for the present study included a set of questions about daily caregiving practices in the home for the study child (e.g., do you play with the child or read to the child most days? Does the child have toys to play with in the home? Are you with the child most of the day and who is with the child when you are not there?).

Measures from the SES questionnaire (Caregiving, Food Security and Nutrition) along with clinic-based measures of anemia and malaria illness were included along with maternal HIV status in a conceptual model of child neurodevelopment depicted in Fig. 1. This conceptual model for the present study guided the statistical analyses described below for the principal study measures and MSEL outcomes.

### Statistical analyses

Although in the published findings of the RCT study, the principal outcome was malaria illness incidence per person year at risk (PYAR) [14, 15], counts of malaria episodes were used as the primary exposure variable in the present analyses. This is because the PYAR measure was possibly compromised due to the aggressive treatment course in response to all bouts of malaria, as reported in the RCT study. This included the inclusion of a mandatory 6-week period of parasite clearance verification for each treatment episode for each child. However, substituting the PYAR measure for simple number of malaria episodes did not change any of the principal findings from the analyses described below.

The principal predictor in the present analyses was incidence of malaria, defined as the number of episodes during the period the chemoprevention was given, and between 2 and 3 years of age after chemoprevention was stopped. The other exposure variable was incidence of anaemia during the study period: the number of incidents of moderate-severe anaemia (haemoglobin <10 gm/dL) during monthly clinic visits during the chemoprevention period (up to 2 years) and during the year following cessation of chemoprevention (between 2 and 3 years of age).

Primary analyses for the first research question about the relation of malaria illness and anaemia with cognitive development was addressed using linear mixed effects (LME) models. The numbers of malaria and anaemia episodes before 2 years, and between 2 and 3 years of age were used as time varying covariates. Other time-varying covariates included age, weight for age z-score (WAZ) and number of breastfeeding days in a time period. Fixed (not time-varying) covariates included trial arm, HIV exposure, sex, socio-economic status (SES), and number of observation days prior to randomization. Height-for-age z scores (HAZ) was also added to the regression models (Tables 2, 3, 4, 5) but was not included in the final analyses because of collinearity issues with WAZ. Substituting HAZ for WAZ did not change any of our principal findings with respect to the relationship between malaria and MSEL outcomes with or without anemia.

The assumption of linearity of the relationship between the Mullen scores and principal exposure variables (number of malaria and anemia episodes) was evaluated using scatterplots. To assess the robustness of findings, sensitivity analyses were performed using several cut-offs in the number of episodes. The results obtained from models with categorical exposure variables were not appreciably different from the results from the models with linear effects.

To assess the effect of exposures and the extent to which the effects of the number of malaria episodes were mediated by the anaemia, three LME models were fit.

Model 1 related the MSEL scores (one at a time) to time period (enrolment to 2 years, or 2–3 years), number of malaria episodes and other covariates described above. The number of anaemia episodes was not included in this model to assess the main effect of the malaria episodes.

Model 2 related the MSEL scores (one at a time) to time period (enrolment to 2 years, or 2–3 years), number of malaria episodes and other covariates. The number of malaria episodes was not included in this model to assess the main effect of the anaemia episodes.

Model 3 related the MSEL scores (one at a time) to time period (enrolment to 2 years, or 2–3 years), number of malaria episodes, number of anaemia episodes, and other covariates. This model assessed the effects of malaria and anaemia episodes over and above each other. To establish mediation, the effect of the number of malaria episodes had to be significant in Model 1. If in Model 3 the effect of the number of malaria episodes was no longer significant, then complete mediation was deemed to occur [21–25]. If the magnitude of the effect of the number of malaria episodes was diminished, but was still significant after the inclusion of anaemia, then partial mediation had occurred. To gauge any effects that may differ according to the HIV status, Models 1–3 were run again separately for the HEU and HUU children. All analyses were performed in SAS 9.4.

## Results

The subgroup of children from the RCT cohorts (HUU or HEU) who were not co-enrolled in the neurodevelopmental observational MSEL study did not differ statistically from the present study samples on any of the descriptive measures in Table 1. In this table, the HEU children did more poorly than the HUU children on the MSEL receptive language and the MSEL composite (cognitive development) measure at 3 years of age, but not 2 years (*P* < 0.01). HEU children had fewer bouts of malaria during both the chemoprevention phase (6 months to 2 years) (*t* = 5.94, *P* < 0.001) and post prevention (2–3 years) (*t* = 3.78, *P* < 0.001). The HEU children also had fewer bouts of anaemia before 2 years of age (*t* = 2.35, *P* = 0.02), and fewer exclusively breastfeeding days both before and during the study (*t* = 7.47, *P* < 0.001) (see Table 1). The HEU children did more poorly than the HUU cohort on the MSEL at 3 years of age (Table 1). They were significantly lower on the MSEL composite cognitive total (*t* = 3.19, *P* = 0.002), receptive language (*t* = 3.07, *P* < 002), and visual reception (*t* = 1.97, *P* < 0.05). The HEU cohort also did more poorly than the HUU children on the MSEL expressive language scale at 2 years of age (*t* = 1.98, *P* < 0.05) (Table 1).

**Table 1:** Descriptive statistics for the study sample for perinatally HIV-unexposed and uninfected (HUU) and exposed and uninfected (HEU)

Characteristic	Perinatally HIV unexposed and uninfected (HUU) N = 325 at 2 years N = 331 at 3 years	Perinatally HIV exposed and uninfected (HEU) N = 143 at 2 years N = 122 at 3 years
	Mean (SD)	Mean (SD)
Age at first MSEL neurodevelopmental assessment (years)	2.20 (0.16)	2.17 (0.16)
Age at second MSEL neurodevelopmental assessment (years)	3.02 (0.06)	3.05 (0.14)
Weight-for-age z-score at first assessment^a^	−0.10 (1.09)*	−0.38 (1.03)
Weight-for-age z-score at second assessment^a^	−1.25 (1.05)	−1.24 (0.98)
Days of observation prior to randomization	22.13 (15.51)	204.39 (107.27)
Days breastfeeding before 2 years of age	634.48 (95.79)***	284.82 (106.76)
Days breastfeeding between 2 and 3 years of age	12.92 (31.42)***	0 (0)
Number of malaria episodes before 2 years of age	7.38 (4.95)	4.27 (4.21)***
Number of malaria episodes between 2 and 3 years	7.93 (3.93)	6.27 (4.19)*
Number of anaemia episodes (Hb < 10 gm/dL) before 2 years	7.10 (5.37)	5.56 (4.97)*
Number of anaemia episodes (Hb < 10 gm/dL) between 2 and 3 years	0.56 (0.76)	0.70 (0.80)
MSEL gross motor (raw score) at 2 years	23.5 (3.28)	23.4 (3.16)
MSEL gross motor (raw score) at 3 years	27.5 (3.14)	27.3 (3.13)
MSEL visual reception (T score) at 2 years	35.0 (9.22)	34.0 (9.65)
MSEL visual reception (T score) at 3 years	31.8 (10.06)	29.6 (10.52)
MSEL fine motor (T score) at 2 years	33.8 (9.24)	33.5 (9.38)
MSEL fine motor (T score) at 3 years	36.5 (7.85)	34.9 (8.68)
MSEL receptive language (T score) at 2 years	41.4 (10.4)	40.9 (10.38)
MSEL receptive language (T score) at 3 years	42.0 (7.71)**	39.4 (8.45)
MSEL expressive language (T score) at 2 years	36.1 (7.91)*	34.5 (7.57)
MSEL expressive language (T score) at 3 years	37.4 (10.77)	37.3 (11.43)
MSEL composite cognitive total (standard score) at 2 years	75.0 (12.04)	73.6 (12.15)
MSEL composite cognitive total (standard score) at 3 years	75.6 (11.94)**	72.0 (10.14)
*Sex*	*N (%)*	*N (%)*
Male	174 (53)	63 (52)
Female	152 (47)	59 (48)
*Treatment arm from 6* *months to 3* *years of age*	*N (%)*	*N (%)*
No chemoprevention (placebo)	85 (25)	33 (27)
Monthly sulfadoxine–pyrimethamine (SP)	85 (26)	26 (21)
Daily trimethoprim–sulfamethoxazole (TS)	80 (24)	29 (24)
Monthly dihydroartemisinin–piperaquine (DP)	81 (25)	34 (28)

* *P* < 0.05; ** *P* < 0.01; *** *P* < 0.001

^a^z scores for this physical growth measure were based on World Health Organization 2013 global norms

Table 2 presents the mean and standard deviation for the number of malaria and anaemia episodes during the year following chemoprevention, and also includes the MSEL average group performance by treatment arm at 2 and 3 years of age. As was the case in the principal publication of RCT results for both cohorts separately [14, 15], DP and TS both resulted in fewer incidences of malaria during chemoprevention treatment compared to SP and the no treatment arm (*F* = 12.47, *P* < 0.001 for HUU; *F* = 9.83, *P* < 0.001 for HEU). These treatment-arm differences in malaria episodes no longer occurred after treatment was stopped at 2 years of age (Table 2). Episodes of anaemia (haemoglobin <10 gm/dL) were also fewer for the DP arm during the chemoprevention phase, but only for the HUU cohort (*F* = 4.90, *P* = 0.002). After cessation of treatment, there were no longer significant differences among treatment arms on episodes of anaemia. There were no significant differences among treatment arms on the MSEL composite total either following chemoprevention (2 years of age assessment) or a year after chemoprevention (at 3 years of age) (Table 2).

**Table 2:** Malaria and anaemia episodes and Mullen Scales of Early Learning (MSEL) scores by chemoprevention trial arm

	Malaria episodes before 2 years of age	Malaria episodes 2–3 years of age	Anaemia episodes before 2 years of age	Anaemia episodes 2–3 years of age	MSEL composite at 2 years of age	MSEL composite at 3 years of age
*RCT trial arm*—*HUU cohort*						
No chemoprevention (placebo)	8.68 (4.80)	7.70 (4.07)	7.95 (5.71)	0.48 (0.77)	76.08 (11.67)	75.15 (11.31)
Daily trimethoprim–sulfamethoxazole (TS)	7.25 (4.74)*	7.77 (3.82)	7.06 (5.37)	0.76 (0.95)	73.32 (12.78)	75.74 (13.74)
Monthly sulfadoxine–pyrimethamine (SP)	8.70 (4.85)	8.19 (4.08)	8.06 (5.48)	0.51 (0.69)	74.79 (11.46)	76.13 (11.03)
Monthly dihydroartemisinin–piperaquine (DP)	4.76 (4.39)***	8.00 (3.83)	5.24 (4.41)**	0.49 (0.66)	75.78 (12.24)	77.04 (11.78)
*RCT trial arm*—*HEU cohort*						
No chemoprevention (placebo)	6.87 (5.09)	6.94 (4.66)	6.36 (6.31)	0.70 (0.88)	73.00 (12.39)	74.06 (10.22)
Daily trimethoprim–sulfamethoxazole (TS)	2.59 (2.51)***	6.21 (4.49)	4.83 (3.70)	0.76 (0.74)	75.56 (13.17)	74.21 (9.90)
Monthly sulfadoxine–pyrimethamine (SP)	5.85 (4.25)	6.27 (3.85)	7.42 (6.02)	0.65 (0.80)	74.45 (12.19)	70.73 (9.36)
Monthly dihydroartemisinin–piperaquine (DP)	2.74 (3.15)***	5.68 (3.77)	4.88 (3.62)	0.68 (0.80)	74.45 (12.19)	69.21 (10.43)

** *P* < 0.01; *** *P* < 0.001

In Table 3 (HUU and HEU combined), malaria episodes were related to MSEL scores at 2 and 3 years of age when not controlling for anaemia episodes (Model 1) and when controlling for anaemia episodes (Model 3). This Table also evaluates the extent to which anaemia episodes (during and after chemoprevention) predict MSEL when not controlling for malaria episodes (Model 2) and controlling for malaria episodes (Model 3). Malaria episodes were significantly related to the MSEL composite score (*P* = 0.02), but not when controlling for anaemia episodes (*P* = 0.13), suggesting that anaemia mediates this relationship. The same is true for the MSEL outcomes of fine motor (*P* < 0.01 to *P* = 0.03) and receptive language (*P* = 0.04 to *P* = 0.15). Similar mediational effects were observed when comparing the relationship of anaemia episodes to MSEL with and without controlling for malaria episodes. This was the case for composite cognitive ability (*P* = 0.01 to *P* = 0.10), visual reception (*P* < 0.01 to *P* = 0.02) and receptive language (*P* = 0.03 to *P* = 0.14) when exploring the malaria and anaemia episode effects (Table 3).

**Table 3:** Malaria and anaemia episodes effects on the Mullen Scales of Early Learning (MSEL) scores for both cohorts combined (HUU and HEU)

MSEL outcome scores	Malaria episodes effects				Anaemia episodes effects			
	Model 1: not controlling for anaemia episodes		Model 3: controlling for anaemia episodes		Model 2: not controlling for malaria episodes		Model 3: controlling for malaria episodes	
	Coef (SE)	*P* value	Coef (SE)	*P* value	Coef (SE)	*P* value	Coef (SE)	*P* value
Composite	−0.22 (0.09)	0.02*	−0.16 (0.10)	0.13	−0.23 (0.09)	0.01**	−0.17 (0.10)	0.10
Visual reception	−0.12 (0.08)	0.12	−0.04 (0.09)	0.63	−0.23 (0.08)	<0.01**	−0.21 (0.09)	0.02*
Fine motor	−0.19 (0.07)	<0.01**	−0.17 (0.08)	0.03*	−0.14 (0.07)	0.07	−0.07 (0.08)	0.42
Receptive language	−0.16 (0.08)	0.04*	−0.12 (0.08)	0.15	−0.17 (0.08)	0.03*	−0.12 (0.09)	0.14
Expressive language	−0.08 (0.08)	0.39	−0.05 (0.08)	0.54	−0.08 (0.08)	0.36	−0.05 (0.09)	0.56
Gross motor T score	−0.01 (0.03)	0.74	0.01 (0.03)	0.86	−0.03 (0.03)	0.19	−0.03 (0.03)	0.21

* *P* < 0.05; ** *P* < 0.01

Trial arm was *not* significantly related to the Mullen scores over and above other variables in the models, notably after accounting for the numbers of malaria and/or anaemia episodes. Over and above the malaria and anaemia exposure variables and other covariates, the HUU versus HEU status variable was only significant for the receptive language (favouring the unexposed). Significant predictors included age (negative association), and time (lower scores at 3 years of age compared to 2 years).

Table 4 presents the effects of the number of malaria and anaemia episodes in statistical Models 1–3 for just the HUU cohort. In this Table, the only MSEL score significantly related to malaria episodes was fine motor, which was mediated by anaemia episodes (*P* = 0.04 to *P* = 0.18). A similar mediational effect was observed when evaluating the relationship between anaemia episodes and MSEL fine motor (*P* = 0.03 to *P* = 0.14). For Table 5 (HEU cohort), malaria episodes were predictive of MSEL cognitive composite as mediated by anaemia episodes (*P* = 0.02 to *P* = 0.10), as well as predictive of receptive language (*P* = 0.01 to *P* = 0.05). For this cohort, anaemia episodes only predicted MSEL visual reception as mediated by malaria episodes (*P* = 0.04 to *P* = 0.13).

**Table 4:** Malaria and anaemia episodes effects on the Mullen Scales of Early Learning (MSEL) scores for the HUU cohort

MSEL outcome scores	Malaria episodes effects				Anaemia episodes effects			
	Model 1: not controlling for anaemia episodes		Model 3: controlling for anaemia episodes		Model 2: not controlling for malaria episodes		Model 3: controlling for malaria episodes	
	Coef (SE)	*P* value	Coef (SE)	*P* value	Coef (SE)	*P* value	Coef (SE)	*P* value
Composite cognitive total	−0.13 (0.11)	0.26	−0.08 (0.12)	0.54	−0.17 (0.11)	0.13	−0.14 (0.12)	0.25
Visual reception	−0.05 (0.09)	0.58	0.01 (0.10)	0.88	−0.17 (0.10)	0.08	−0.18 (0.11)	0.10
Fine motor	−0.16 (0.08)	0.04*	−0.12 (0.08)	0.18	−0.19 (0.09)	0.03*	−0.14 (0.10)	0.14
Receptive language	−0.06 (0.09)	0.48	−0.03 (0.09)	0.74	−0.11 (0.09)	0.20	−0.10 (0.10)	0.30
Expressive language	−0.04 (0.09)	0.61	−0.04 (0.10)	0.67	−0.04 (0.09)	0.65	−0.02 (0.11)	0.82
Gross motor T score	0.01 (0.03)	0.71	0.02 (0.03)	0.48	−0.02 (0.03)	0.43	−0.03 (0.03)	0.32

* *P* < 0.05; ** *P* < 0.01

**Table 5:** Malaria and anaemia episodes effects on the Mullen Scales of Early Learning (MSEL) scores for the HEU cohort

Mullen outcome scores	Malaria episodes effects				Anaemia episodes effects			
	Model 1: not controlling for anaemia episodes		Model 3: controlling for anaemia episodes		Model 2: not controlling for malaria episodes		Model 3: controlling for malaria episodes	
	Coef (SE)	*P* value	Coef (SE)	*P* value	Coef (SE)	*P* value	Coef (SE)	*P* value
Composite	−0.41 (0.18)	0.02*	−0.32 (0.19)	0.10	−0.32 (0.17)	0.06	−0.19 (0.19)	0.32
Visual reception	−0.26 (0.16)	0.10	−0.14 (0.18)	0.41	−0.35 (0.17)	0.04*	−0.28 (0.18)	0.13
Fine motor	−0.22 (0.14)	0.13	−0.27 (0.16)	0.09	0.00 (0.14)	0.99	0.12 (0.16)	0.46
Receptive language	−0.40 (0.15)	0.01**	−0.34 (0.17)	0.05*	−0.30 (0.16)	0.07	−0.15 (0.18)	0.41
Expressive language	−0.16 (0.15)	0.28	−0.12 (0.17)	0.47	−0.15 (0.16)	0.33	−0.10 (0.17)	0.57
Gross motor T score	−0.06 (0.05)	0.21	−0.04 (0.05)	0.42	−0.06 (0.05)	0.23	−0.04 (0.05)	0.46

* *P* < 0.05; ** *P* < 0.01

## Discussion

Within the context of a rigorous RCT study in a high-malaria-exposure area in rural eastern Uganda, strong evidence is presented that repeated episodes of malaria illness, as mediated by anaemia, can diminish cognitive development in very young children. In the present findings, malaria chemoprevention during early childhood is not directly related to child neurodevelopment. However, malaria episodes during and after chemoprevention are significantly predictive of both cognitive and motor development at 2 and 3 years of age. This study is the first to document within the rigorous context of an RCT study that early uncomplicated malaria illness before 2 and 3 years of age can diminish cognitive development in very young African children. Furthermore, this relationship is at least partially mediated by anaemia. However, another form of analysis that might have been used to better understand the mediating effects of anemia is structural equation modeling. Future work could employ this statistical method as well.

HIV exposure resulted in lower MSEL scores on composite cognitive performance at 3 years of age, especially receptive and expressive language. MSEL differences were observed at 2 years of age only at the 0.05 significance level on Expressive Language (*P* < 0.05). These differences may have been due to differing levels of sensitivity of these scales to the developmental effects of HIV-exposure in this setting, depending on which developmental domains blossom most at a given year of age. For example, MSEL language development differences for HEU children compared to HUU children in rural Uganda has been previously observed in the pre-school age years [26].

It should also be noted that the relationship between episodes of malaria illness and MSEL composite cognitive and receptive language performance for the HEU cohort was more robust than for the HUU cohort in the present study (comparing Tables 4, 5), when the MSEL outcomes were considered at 3 years of age. Cognitive development risk from malaria and anaemia seems to be greater in HEU children, especially as they enter into the critical preschool-age years at 3 years of age. However, the clinical significance of the statistically significant relationships observed in the present study between malaria illness and MSEL developmental outcomes is uncertain. This is because true Uganda-based norms are missing for the MSEL, and only American-based norms are available for standardizing performance on the basis of age and gender.

Despite this more robust relationship, the overall number of episodes of malaria illness and anaemia during and after chemoprevention was lower for the HEU than for the HUU cohorts (Table 1). This is likely due to the fact that in the RCT study within which this observational study was embedded, the HEU children were maintained on TS while nursing, rather than being weaned and randomized to the chemoprevention treatment arms at 6 months of age as were the HUU children. Both TS and DP chemoprevention significantly reduced the number of malaria episodes compared to the SP and placebo treatment arms [14, 15, 27].

Although a significant relationship was observed between episodes of malaria illness and neurodevelopmental outcomes in the present study findings, significant neurodevelopmental differences were not observed among chemoprevention treatment arms, either during or after the treatment phase. This might be partly due to questionable adherence to the treatment arm regimens on the part of the mothers of the children, as witnessed by the results of monitoring of DP blood levels in the RCT trial [14, 15]. Chemoprevention treatment adherence was mostly monitored by self-report on the part of the mothers during their monthly clinic visits. Such adherence issues would also pertain to the use of prophylactic antibiotics and micronutrient supplements provided for participants during the study period.

Finally, socio-economic and educational status can impact on both the reliable use of malaria chemoprevention medications and quality of housing in terms of night-time exposure of study children to mosquitoes [28]. In the previous study of HEU children in Tororo, SES, maternal educational level, nutritional quality, housing quality, and maternal depression were all related to child neurodevelopment independent of malaria episodes per se [18]. Duration of breast feeding is related to morbidity for both the HEU and HUU children in the study cohorts [29]. These important factors related to child development may not have been equally distributed across chemoprevention treatment arms in this observational study within the larger RCT study.

The present study findings support other studies with school-age children that conclude that repeated episodes of malaria are associated with poor cognitive scores and low academic performance [9, 30]. Even asymptomatic positive malaria parasitaemia led to poorer cognitive performance among school-age children at the Tororo study site [31]. Unfortunately, the relationship between asymptomatic positive parasitaemia and MSEL measures of neurodevelopment could not be evaluated in the present study. Whenever children developed a fever and had a positive blood smear the day before or at their monthly clinic visit, they were treated with AL and monitored for parasite clearance following treatment.

Because convenience sampling was used to enroll the original HEU cohort of 200 and the HUU cohort of 400 infants at 4–5 mo of age from the Tororo District Hospital Maternal and Child Health Clinic their birth records were incomplete. Therefore, premature and low-birth weight children could not be excluded and this may have led to developmental delays that could compromise the sensitivity of the MSEL outcomes to the effects of malaria illness frequency and anaemia in the present samples. HEU children in this study area are at significantly greater risk for premature birth and complications, and shorter duration of breast feeding [32]. These risk factors can lead to developmental delays at measured by the MSEL [26]. Maternal depression for mothers with HIV can also affect MSEL outcomes for HEU children, and this was not measured for mothers in the present study [33]. This could also obscure the relationship between malaria or anaemia and MSEL outcomes in the present analyses and serve as a study limitation.

Beyond LLIN distribution programmes, little has been done for very young children in Africa to protect them against malaria. In rural households in Tororo District which have been provided with LLINs, children can still have as high as 310 infectious bites per year with a malaria-positive parasite prevalence rate of 28.7 % [28]. In the present study cohorts, where LLINs and prompt effective treatment were provided, the risk of complicated malaria and anaemia was extremely low. However, incidence of malaria illness was still high with the present standard of care (SP) (malaria illnesses > seven times/year) and increased as soon as more effective chemoprevention (DP and TS) was discontinued. This suggests that more is needed in early malaria prevention than simply improved LLIN distribution. This includes preventing malaria exposure for mothers during pregnancy, especially mothers with HIV [34].

In the longer term, malaria vaccines may have an important role in protecting this important section of the community from malaria. Regardless of the control approach selected, it is important that a comprehensive and aggressive community-wide approach to controlling malaria be incorporated into every public health programme in malaria-endemic regions, as part of the ongoing effort to enhance the health of African school-age children [30].

## Conclusions

Early bouts of malaria illness can diminish cognitive development in very young children. These effects of malaria illness on cognition are partly mediated by anaemia, and cognitive development risk from malaria and anaemia seems to be greater in HEU children. This is the case even with children and caregivers who are provided with free and effective medical care and nutritional support in the first years of life. The high level of malaria transmission and the host of other risk factors in such low-resource environments can still overwhelm such medical and nutritional support, even when provided in a conscientious manner in such settings.

## Availability of supporting data

We will be able to download our de-identified datasets and data definition dictionary from our university data management archive to make them available to other research groups upon request. We will require a collaborative agreement from such groups before making such datasets available, as approved through the Intellectual Property Office at Michigan State University. See http://newsroom.msu.edu/site/indexer/3004/content.htm for an overview of this office and http://oip.msu.edu/documents/CDA2001_002.pdf for an example of such an agreement. In addition, we will ask for final approval of all publications from other research groups that include our study data.

## Abbreviations

AL: artemether–lumefantrine
ANCOVA: analysis of covariance
DP: dihydroartemisinin–piperaquine
HAART: highly active anti-retroviral therapy
HAZ: height-for-age z score
HIV: human immunodeficiency virus
IPT: intermittent preventive treatment
IRB: Institutional Review Board
LLIN: long-lasting insecticide-treated bed net
LME: linear mixed-effects model
MSEL: Mullen Scales of Early Learning
HEU: perinatally HIV-exposed and uninfected
HUU: perinatally HIV-unexposed and uninfected
PYAR: person-year at risk
SES: socio-economic status
SP: sulfadoxine–pyrimethamine
TS: trimethoprim–sulfamethoxazole
UNCST: Ugandan National Council for Science and Technology
WAZ: weight-for-age adjusted Z score
WHO: World Health Organization
